# Long-term safety of human papillomavirus vaccination in relation to early pregnancy outcomes: registry-based follow-up of a community-randomized trial

**DOI:** 10.1093/eurpub/ckag102

**Published:** 2026-08-12

**Authors:** Tiina Koivisto, Kaisa Taavela, Tiina Eriksson, Dan Apter, Matti Lehtinen, Karolina Louvanto

**Affiliations:** Faculty of Medicine and Health Technology, Tampere University, Tampere, Finland; Wellbeing Services County of Pirkanmaa, Tays Research Services, Tampere University Hospital, Tampere, Finland; Department of Obstetrics and Gynecology, Tampere University Hospital, Tampere, Finland; Faculty of Medicine and Health Technology, Tampere University, Tampere, Finland; Wellbeing Services County of Pirkanmaa, Tays Research Services, Tampere University Hospital, Tampere, Finland; Department of Obstetrics and Gynecology, Tampere University Hospital, Tampere, Finland; Department of Obstetrics and Gynecology, Seinäjoki Central Hospital, Seinäjoki, Finland; FICAN-Mid, Tampere, Finland; VL-Medi Clinical Research Center, Helsinki, Finland; Department of CLINTEC, Karolinska Institute, Stockholm, Sweden; Faculty of Medicine and Health Technology, Tampere University, Tampere, Finland; Department of Obstetrics and Gynecology, Tampere University Hospital, Tampere, Finland

## Abstract

Despite the demonstrated safety of human papillomavirus vaccines, concerns have arisen regarding potential associations of human papillomavirus vaccination on reproductive health. The objective of this registry-based study was to compare overtime association of vaccination and early pregnancy outcomes. Twenty thousand four hundred twenty-six women born in 1992–1995, who participated in a Finnish community-randomized human papillomavirus vaccination trial, and age- and community-aligned unvaccinated controls born in 1990–1991 (*n* = 19 473) were followed up for 11 years. Data was obtained from the nationwide Finnish Care Register for Health Care. Incidence rates of adverse early pregnancy outcomes were compared between the human papillomavirus vaccinated and unvaccinated group. The mean age at first abnormal pregnancy result was 23.5 years in the human papillomavirus vaccinated group and 22.2 years in the unvaccinated group. 1.6% of human papillomavirus vaccinated women experienced a miscarriage compared with 5.3% of unvaccinated controls (*P *< .0001). No miscarriages occurred in close temporal proximity to vaccination. Rates of ectopic pregnancies were low and comparable across all groups (0.6–1.0%). Among women with a history of abnormal pregnancy outcomes, 55.6% of human papillomavirus vaccinated individuals had at least one live birth, compared with 70.3% of unvaccinated women. Human papillomavirus vaccination was not associated with an increased risk of miscarriage or other adverse early pregnancy outcomes. These results provide reassurance that human papillomavirus vaccination is safe with respect to reproductive outcomes, supporting its continued use in adolescent and young adult populations.

## Introduction

Human papillomavirus (HPV) infection is a well-established etiological factor for cervical cancer [1]. To prevent HPV infections and related cancers, national HPV vaccination programs have been implemented in more than 100 countries [2]. These vaccines have demonstrated high efficacy in preventing HPV infections and related diseases, thereby significantly reducing the global burden of cervical cancer and other HPV-associated conditions [3–7].

Although HPV infection is best known for its role in cancer development, it and associated treatments have also been linked to adverse pregnancy outcomes, including spontaneous abortion, preterm birth, premature rupture of membranes, intrauterine growth restriction, low birth weight and fetal death [8–12]. Consequently, HPV vaccination may also have beneficial effects on pregnancy outcomes by preventing HPV infections. Some studies have reported a modest reduction in preterm birth rates among HPV-vaccinated women [13, 14].

Evidence regarding the safety of HPV vaccination in relation to reproductive events has been mixed. Earlier studies reported conflicting findings concerning the safety of HPV vaccination during early pregnancy [15–17]. However, recent meta-analyses and systematic reviews indicated that there is no increased risk of stillbirth, small-for-gestational-age infants, preterm birth, or congenital anomalies among women vaccinated during pregnancy or the peri-conceptional period [18, 19]. Similarly, no consistent association has been found between HPV vaccination and an increased risk of miscarriage [16, 17, 20, 21].

Given these uncertainties, further research is needed to clarify the relationship between HPV vaccination and reproductive outcomes. The aim of this study was to compare the incidence of miscarriages and other abnormal early pregnancy events between HPV-vaccinated and unvaccinated women.

## Methods

### Study cohort

This longitudinal cohort study includes 20 426 women from the 1992–1995 birth cohorts, who originally participated in a community-randomized HPV vaccination trial initiated in 2007 across 33 municipalities in Finland, and of a reference cohort of 19 473 unvaccinated, age- and community-aligned women born in 1990–1991 [22, 23]. During the randomized HPV vaccination trial, participants received either the bivalent AS04-adjuvanted HPV16/18 vaccine (later in the text “HPV-vaccinated”) or the hepatitis B vaccine (later in the text “HBV-vaccinated”) at ages 12–15 between 2007–2009, as previously described [23]. Study nurses administered the vaccines intramuscularly following a three-dose-schedule (0, 1, and 6 months). At 18.5 years of age, trial participants were offered cross-vaccination with the alternative vaccine that they had not previously received. Participants who received the HPV vaccination at that time are hereafter referred to as “cross-vaccinated.”

Age-aligned registry data after the first HPV vaccine dose was obtained from the nationwide Finnish Care Register for Health Care to compare early pregnancy complication rates between HPV-vaccinated and unvaccinated individuals. The registry data included information according to the International Classification of Diseases, 10th Revision (ICD-10) codes for early pregnancy failure or loss (O00: Ectopic pregnancy, O01: Hydatidiform mole, O02: Other abnormal products of conception, and O03: Spontaneous abortion), as well as procedural codes related to healthcare visits (e.g. ultrasounds, surgical interventions). Live birth data were received from the Finnish Medical Birth Registry, which includes data on live births, and on stillbirths of fetuses with a birth weight of at least 500 g or with a gestational age of at least 22 weeks.

The follow-up period was 11 years for all individuals, covering ages 16–26 or 17–27 depending on the birth year. For HPV-vaccinated individuals born in 1992–1993, the follow-up extended from 1 January 2009–31 December 2019; for those born in 1994–1995, from 1 January 2011–31 December 2021; and for unvaccinated individuals born in 1990–1991, from 1 January 2007–31 December 2017.

The original community-randomized HPV vaccination trial received approval from the Pirkanmaa Hospital District Ethical Review Board (R07113M/12/6/2007). The Finnish Social and Health Data Permit Authority (Findata) authorized the linkage of registry data for unvaccinated individuals (THL/5238/14.02.00/2020 and THL/903/14.02.00/2022).

### Statistical analyses

The primary comparison was between cross-vaccinated and unvaccinated individuals. The vaccination age 18.5 was selected as the primary focus of examination, as it is closer to the potential age of family planning than would be the case for individuals at ages 12–15. Differences between the HPV- and HBV-vaccinated compared to unvaccinated group were also examined.

The registry data included information on the dates of HPV vaccinations, miscarriages and other abnormal early pregnancy events, and relevant diagnosis codes (ICD-10 codes O00, O01, O02 and O03), as well as procedure codes used during healthcare visits (e.g. ultrasounds, surgical procedures).

Early pregnancy losses and complications (O00-O03) were included in the analyses. Specifically, the spontaneous abortion (O03) and other abnormal products of conception (O02) were combined to form the main “miscarriage” group. Live birth data was used to determine whether individuals who experienced miscarriages also had pregnancies.

The Finnish Care Register for Health Care documents all patient visits, diagnoses, and procedure codes. To ensure the diagnostic accuracy for each abnormal early pregnancy event (miscarriage, ectopic, or molar pregnancy), we used the latest registered ICD-10 code across all related visits, avoiding inclusion of preliminary diagnoses later revised. To prevent duplicate registration, diagnosis and procedure codes recorded within 90 days of an initial abnormal early pregnancy-related visit were excluded. Any codes recorded after the 90-day window were manually reviewed by exploring diagnosis and possible procedural codes to confirm whether they represented a new, independent event.

Incidence rates of abnormal early pregnancy events were calculated per 10 000 person-years for HPV-vaccinated, HBV-vaccinated, cross-vaccinated and unvaccinated individuals, with follow-up extending up to ages 26 or 27, depending on birth year. Multiple miscarriages and ectopic pregnancies among the same individuals were included in the analyses. Miscarriage, ectopic pregnancy and molar pregnancy numbers were further examined across three age groups: 15–19, 20–24, and 25–27 years. Additionally, miscarriage incidence rates were assessed at five time points (6, 12, 18, 24, and >24 months) for cross-vaccinated, following the first HPV vaccine dose at 18.5 years. Comparable time-point analyses were conducted for unvaccinated, age- and community-aligned individuals, with follow-up commencing at 18.5 years.

In all tables, counts less than three are denoted as (<3) to ensure confidentiality. The health registry data protection regulations prohibit the publishing of cell sizes less than three. Rates, with 95% confidence intervals, were calculated using the Rothman Greenland method [24] to compare the incidence rates between groups. All statistical analyses were performed using SPSS IBM Statistics 29.0, and a two-sided *P* value <.05 was considered statistically significant.

## Results

In this 11-year follow-up, early pregnancy outcomes were compared among women who participated in a community-randomized HPV vaccination trial, and among age- and community-aligned unvaccinated controls. Overall, 1.6% of HPV-vaccinated, 1.3% of cross-vaccinated (received three doses of the HPV16/18 vaccine at age 18.5), 1.4% of HBV-vaccinated and 5.3% of unvaccinated individuals had a recorded miscarriage during 11-year follow-up period (Table 1). The difference between cross-vaccinated and unvaccinated women was statistically significant (*P* < .0001). Mean age at the time of first abnormal early pregnancy event (miscarriage or ectopic pregnancy or molar pregnancy) was 23.5 years among HPV-vaccinated, 23.4 years among HBV-vaccinated, 24.1 years among cross-vaccinated and 22.2 years among unvaccinated. The number of ectopic pregnancies was low and comparable across all groups, ranging from 0.6% to 1.0%. Due to the very small number of molar pregnancies, exact figures could not be published in accordance with data protection regulations. Other baseline characteristics were not available from the Finnish Care Register for Health Care.

**Table 1.** Comparison of early pregnancy complications and live births among women vaccinated against human papillomavirus or against hepatitis B virus*, and among age- and community-aligned unvaccinated** women over an 11-year follow-up in Finland during years 2007–2021

	**HPV** *n* = 12 345	**Cross-vaccinated** *n* = 4549	**HBV** *n* = 3532	**Unvaccinated** *n* = 19 473
Mean age at the time of 1st abnormal early pregnancy event^a^, years (±SD)	23.5 (±2.6)	24.1 (±2.2)	23.4 (±2.7)	22.2 (±2.9)
Miscarriages^b^, n (%)	199 (1.6)	59 (1.3)	50 (1.4)	1040 (5.3)
Ectopic pregnancies, n (%)	74 (0.6)	32 (0.7)	35 (1.0)	155 (0.8)
Any abnormal pregnancy^a^, n (%)	275 (2.2)	92(2.0)	85 (2.4)	1214 (6.2)
Live births among individuals with a history of ≥1 abnormal early pregnancy event^a^, n (%).	153 (55.6)	41 (44.6)	42 (49.4)	853 (70.3)

Abbreviations: HPV = Human papillomavirus, HBV = Hepatitis B virus, SD = Standard deviation.

* Women born in 1992–1995, who participated in a randomized HPV vaccination trial, during which participants received either the bivalent HPV16/18 vaccine (HPV) or the hepatitis B vaccine (HBV) at ages 12–15. Participants who had received the hepatitis B vaccine during the initial randomization, subsequently were able to receive cross-vaccination with the HPV vaccine at 18.5 years (Cross-vaccinated).

** Unvaccinated: Age- and community-aligned unvaccinated reference cohort born in 1990–1991.

a Any early pregnancy losses and complications of Ectopic pregnancy (O00), Hydatidiform mole (O01), Other abnormal products of conception (O02), or Spontaneous abortion (O03).

b Including both Other abnormal products of conception (O02) and Spontaneous abortion (O03).

Linkage to the Finnish Birth Register enabled assessment of live births among individuals with a history of abnormal pregnancy outcomes. Among HPV-vaccinated women with at least one abnormal pregnancy outcome (miscarriage, ectopic pregnancy, or molar pregnancy), 55.6% (153/275) had at least one live birth. The corresponding proportions were 49.4% (42/85) among HBV-vaccinated women, 44.6% (41/92) among cross-vaccinated women, and 70.3% (853/1214) among unvaccinated women. The differences in live birth rates between HPV- or HBV-vaccinated and unvaccinated women, who had had abnormal pregnancy outcomes, were statistically significant (*P* < .00001 and *P* < .00006, respectively).

Incidence rates of abnormal pregnancy outcomes were assessed across three age groups among vaccinated and unvaccinated individuals (Table 2). No significant differences were observed between the vaccinated groups in any age category. The highest miscarriage incidence rates were observed in the oldest age group (25–27 years), ranging from 7 to 8 per 10 000 person-years (95%CI 5–10). These rates were notable lower than those observed in the unvaccinated group, which had a miscarriage incidence rate of 14 per 10 000 person-years (95%CI 13–16).

**Table 2.** Incidence rates of miscarriages^a^ and ectopic pregnancies across three age groups among women vaccinated against human papillomavirus or against hepatitis B virus*, and among age- and community-aligned unvaccinated** women over an 11-year follow-up in Finland during 2007–2021

	**HPV** (*n* = 12 345)		**Cross-vaccinated** (*n* = 4549)		**HBV** (*n* = 3532)		**Unvaccinated** (*n* = 19 473)	
	*n* (%)	Rate/10 000 person years (95% CI)^a^	*n* (%)	Rate/10 000 person years (95% CI)^a^	*n* (%)	Rate/10 000 person years (95% CI)^a^	*n* (%)	Rate/10 000 person years (95% CI)^a^
**Miscarriages** ^b^								
15–19 years	13 (0.1)	1 (0.6–2)	< 3^c^	NC	3 (0.08)	0.8 (0.2–2)	222 (1.1)	10 (9–12)
20–24 years	82 (0.7)	6 (5–7)	24 (0.5)	5 (3–7)	20 (0.6)	5 (3–8)	510 (2.6)	24 (22–26)
25–27 years	104 (0.8)	8 (6–9)	34 (0.7)	7 (5–10)	27 (0.8)	7 (5–10)	308 (1.6)	14 (13–16)
Total	199 (1.6)	15 (13–17)	< 61 (∼1.3)	∼12 (9–15)	50 (1.4)	13 (10–17)	1040 (5.3)	49 (46–52)
**Ectopic pregnancies**								
15–19 years	10 (0.08)	0.7 (0.4–1)	4 (0.09)	0.8 (0.3–2)	5 (0.1)	1 (0.5–3)	20 (0.1)	1 (0.6–1.4)
20–24 years	42 (0.3)	3 (2–4)	10 (0.2)	2 (1–4)	15 (0.4)	4 (2–6)	91 (0.5)	4 (3–5)
25–27 years	22 (0.2)	2 (1–2)	18 (0.4)	4 (2–6)	15 (0.4)	4 (2–6)	44 (0.2)	2 (2–3)
Total	74 (0.6)	5 (4–7)	32 (0.7)	6 (5–9)	35 (1.0)	9 (6–13)	155 (0.8)	7 (6–8)

Abbreviations: HPV = Human papillomavirus, HBV = Hepatitis B virus, CI = Confidence interval, NC = Not computable.

* Women born in 1992–1995, who participated in a randomized HPV vaccination trial, during which participants received either the bivalent HPV16/18 vaccine (HPV) or the hepatitis B vaccine (HBV) at ages 12–15. Participants who had received the hepatitis B vaccine during the initial randomization, subsequently were able to receive cross-vaccination with the HPV vaccine at 18.5 years (Cross-vaccinated).

** Unvaccinated: Age- and community-aligned unvaccinated reference cohort born in 1990–1991.

a Rothman Greenland method.

b Including both Other abnormal products of conception (O02) and Spontaneous abortion (O03).

c Health registry data protection regulations prohibit publishing of cell sizes less than three.

Ectopic pregnancy incidence rates showed overlapping confidence intervals across all groups and age categories. Among cross-vaccinated individuals rates ranged from 0.8 to 4 per 10 000 person-years, and among HPV-vaccinated individuals from 0.7 to 3 per 10 000 person-years, with highest rates observed in older age groups. In the unvaccinated group, the ectopic pregnancy rate ranged from 1 to 4 per 10 000 person-years, suggesting no clear difference in risk between vaccinated and unvaccinated cohorts.

The time from vaccination to the first miscarriage was evaluated across groups. More specifically, miscarriage rates were examined with increasing intervals between vaccination and pregnancy, beginning with six-month time frames. The primary evaluation was conducted in the cross-vaccinated group, as no registered miscarriages occurred within the first six years following the earlier HPV or HBV vaccination schedule (age of 12–15). For the unvaccinated comparison group, time frames were aligned to start from age 18.5 years onwards. During the first 24 months of follow-up, the cross-vaccinated cohort experienced four registered miscarriages (none of them occurring in the first six months), compared to 146 in the age-aligned unvaccinated comparison group.

## Discussion

In this large, population-based cohort with 11 years of age-aligned follow-up, HPV vaccination was not associated with increased risk of early pregnancy complications. Women vaccinated against HPV, whether during adolescence or as cross-vaccination in late adolescence, had lower rates of miscarriage and similar rates of ectopic pregnancy than unvaccinated, age- and community-aligned controls. These findings provide reassurance that HPV vaccination does not appear to be adversely associated with subsequent reproductive outcomes.

Our findings are in line with earlier studies demonstrating no increased risk of miscarriage or other early pregnancy complications following HPV vaccination [20, 21]. In our study, individuals vaccinated with HPV (or HBV vaccination) had overall lower miscarriage rates compared with their age-aligned unvaccinated counterparts, which is consistent with results from prior registry-based investigations and clinical trials. The similarity in miscarriage rates across all vaccinated groups, regardless of vaccine type, is consistent with the hypothesis that trial participants may share similar baseline health behaviors [25], as supported by previous studies reporting no evidence that HPV vaccination elevates miscarriage risk or negatively impacts fertility [19, 26].

The mean ages at miscarriage were slightly higher among HPV-vaccinated (23.5 years) and cross-vaccinated individuals (24.1 years) compared with unvaccinated women (22.2 years). In a large Nordic registry-based study, the lowest risk of miscarriage was observed among women aged 25–29 years (10%), with the risk increasing markedly thereafter [27]. In our study, absolute miscarriage rates in the corresponding age range were substantially lower among vaccinated women (approximately 1%). A growing tendency for miscarriage with increasing age was likewise observed across all vaccinated groups in our study. Surprisingly, among unvaccinated individuals, the miscarriage rate was highest in the 20–24 age group (2.6%) and decreased in the 25–27 age group (1.6%). Although miscarriage risk is generally known to rise with age, these age groups are still relatively close each other, so a clear age-related trend may not be detectable between such narrow and young age categories. The increase in miscarriage risk would be expected to become more apparent after around age 30, and especially at older maternal ages. Regarding the primary comparison groups in our study, the cross-vaccinated group had lower overall miscarriage rate than the unvaccinated group (∼1.3% vs 5.3%), which likely reflects the selection bias (discussed further below) rather than a biological protective effect.

Linkage with the Finnish Medical Birth Register showed that approximately half of HPV-vaccinated women who had experienced an abnormal pregnancy outcome also had at least one liveborn child during the 11-year follow-up period, extending up to ages 26–27. The higher number of live births observed in the unvaccinated group could potentially reflect selection bias rather than a physiological difference. As previously suggested, trial participants can differ from the general population in health-seeking behaviors and socio-demographic factors. In this context, it is possible that vaccinated individuals were more likely to use contraception consistently or prioritize education and career, which may have led to a later start for family planning [25]. Furthermore, given that the mean age at first childbirth in Finland was 30.0 years in 2021 [28], our findings suggest normal reproductive potential among vaccinated individuals and support the absence of adverse association with fertility.

Some earlier reports have raised concerns about the possibility of increased miscarriage risk shortly after vaccination [15]. However, in our study, no miscarriage events occurred in close temporal proximity to HPV vaccination, and no early miscarriages were observed during the first six years following the adolescent vaccination schedule. In the original vaccination trial, pregnancy was ruled out before vaccination, ensuring that participants were not vaccinated while pregnant. Although our registry data could not verify true vaccination status for unvaccinated controls (whether they may have obtained HPV vaccination privately), prior evidence indicates that inadvertent HPV vaccination during pregnancy does not increase the risk of adverse outcomes [19, 21, 29]. Together, these findings reinforce the growing body of evidence demonstrating that HPV vaccination is not associated with any observed increase in risk of miscarriage or other adverse pregnancy outcomes, even when administered in late adolescence.

A key strength of our study lies in its large, nationally representative cohort and the use of high-quality registry data. In Finland healthcare visits and diagnoses are comprehensively recorded, ensuring high validity of the Finnish Care Register for Health Care. The use of individual-level linkage across multiple nationwide registries allowed long-term, unbiased follow-up of both vaccinated and unvaccinated individuals.

Nevertheless, some limitations should be acknowledged. Selection bias is possible, as participants in the original HPV vaccination trial may have differed from unvaccinated women in health awareness, lifestyle habits, or other background characteristics. Such differences could potentially have influenced the observed rates of miscarriages. Lifestyle factors, such as smoking, alcohol use, and body mass index, are known to influence miscarriage risk [30], but were not available in our registry data. We also lacked information on the possible use of contraception. However, trial protocols, including routine inquiries by study nurses regarding contraceptive use and pregnancy, may have influenced family planning behaviors among participants [25]. Such trial-related factors likely contributed to the lower pregnancy and miscarriage rates observed in the vaccinated groups compared to the general population controls. We consider the age-aligned follow-up a strength of this study, as it enabled comparison with unvaccinated counterparts from the same communities where the vaccinated individuals lived. However, differences in follow-up timing across calendar years between the groups may have affected the results due to changes in secular trends. It should also be noted that while the accuracy of diagnostic coding in Finnish registers is generally high, preliminary diagnoses (e.g. suspected ectopic pregnancy) may occasionally be revised during follow-up. To minimize this risk, all cases with repeated healthcare visits within 90 days were manually reviewed to ensure accurate classification of independent pregnancy loss events. Any remaining misclassifications are unlikely to have differed between study groups and thus should not have affected the observed associations.

In this large, long-term, registry-based cohort study, HPV vaccination was not associated with an increased risk of miscarriage or other early pregnancy complications. These findings provide further reassurance on the reproductive safety of HPV vaccination, both when administered in early adolescence and as cross-vaccination in late adolescence. Continued surveillance and future studies incorporating detailed lifestyle and clinical data will help further refine our understanding of HPV vaccination and reproductive health outcomes.

## Data Availability

Individual participant data that underlie the results reported in this article, after de-identification, will be available for researchers who provide a methodologically sound proposal with achievable aims. Proposals should be directed via e-mail to Dr. Matti Lehtinen (matti.antero.lehtinen@gmail.com); data requestors will need to sign a data access agreement. Key points In earlier studies, HPV infection has been linked to adverse pregnancy outcomes but the association between HPV vaccination and reproductive events remains unclear. In this study, HPV vaccination was not associated with an increased risk of miscarriage or other early pregnancy complications, providing further reassurance on its reproductive safety. These study results reinforce the growing body of evidence regarding the safety of HPV vaccination in relation to early adverse reproductive events, both when administered in early or late adolescence.
